# Spread of the non-native anemone *Anemonia alicemartinae* Häussermann & Försterra, 2001 along the Humboldt-current large marine ecosystem: an ecological niche model approach

**DOI:** 10.7717/peerj.7156

**Published:** 2019-07-04

**Authors:** Javier Pinochet, Reinaldo Rivera, Paula E. Neill, Antonio Brante, Cristián E. Hernández

**Affiliations:** 1 Departamento de Zoología, Facultad de Ciencias Naturales y Oceanográficas, Universidad de Concepción, Chile; 2 Ecología Evolutiva y Filoinformática, Departamento de Zoología, Facultad de Ciencias Naturales y Oceanográficas, Universidad de Concepción, Concepción, Chile; 3 Colegio Almondale Lomas, Concepción, Chile; 4 Departamento de Ecología, Facultad de Ciencias, Universidad Católica de la Santísima Concepción, Concepción, Chile; 5 Centro de Investigación en Biodiversidad y Ambientes Sustentables (CIBAS), Universidad Católica de la Santísima Concepción, Concepción, Chile

**Keywords:** Marine invasion, Chilean coast, Human-mediated vector, Range expansion, Marine transport

## Abstract

The geographical expansion of invasive species depends mainly on its dispersal potential, and the abiotic and biotic factors affecting it. Knowing the invasive dynamic of non-native species, as well as its behavior at different natural or anthropogenic scenarios, is fundamental for planning conservation management policies and control plans. The invasive sea anemone *Anemonia alicemartinae* in habits from the north (18°S) to the south-central (36°S) coast of Chile and its distribution range has expanded by approximately 1,928 km in the last 50 years. Previous works have proposed that human-mediated southward transport associated with regional-scale maritime activities could explain its rapid spread. To evaluate this hypothesis, we used ecological niche models (ENM) to evaluate the potential colonization of the southernmost area of South America. Additionally, we conducted a post hoc analysis to evaluate the relationship between the prediction of the ENM and human activity measured as the number of landings of ships in ports. The models were built based on presence records of *A. alicemartinae*, and oceanographic variables. Results showed that sea surface salinity and annual sea surface temperature (variance) are the best predictor variables to explain the distribution of *A. alicemartinae*. There was a positive and significant relationship between the geographical distribution of the sea anemone predicted by the ENM and the number of landings, as a proxy of anthropogenic activity. The most susceptible areas to invasion were those that showed the highest variability in both oceanographic predictors. These areas included the Biobío region, Chiloé´s inland sea, Aysén, and Chacabuco regions, which together comprise two biogeographical provinces. These results sustain the proposed hypothesis and, overall, the results suggest that along with the characteristics of the life history of *A. alicemartinae*, oceanographic conditions and maritime transport as vector contribute to the southern range expansion of this invasive cryptogenic species in the Humboldt-current large marine ecosystem.

## Introduction

Numerous studies indicate that the geographic range (or range limits) of invasive species depends on the dispersive phase, which can be affected by abiotic (e.g., biogeographical barriers or currents) and biotic (e.g., predation, competition, facilitation) factors (Levine, Adler & Yelenik, 2004; Arim et al., 2006; Döge, De Oliveira & Tidon, 2015). Additionally, anthropogenic activities can significantly affect the movement and dispersal of invaders (Seabloom et al., 2006; Molnar et al., 2008). For example, invasive marine species can be intentionally transported for aquaculture, ornamental interests, or accidentally transported, such as via attachment to ship hulls, in ballast water, or with exported products (Neill & Arim, 2011). Moreover, under the climate change scenario the probability of establishment and invasion of non-native species could increase, dynamically changing its current distribution (Molnar et al., 2008; Occhipinti-Ambrogi & Galil, 2010; Ojaveer et al., 2015). Thus, knowing the geographic distribution of invasive species, as well as its temporal dynamics given different natural and anthropic scenarios, is important for generating mitigation plans, large-scale biodiversity management and conservation policies (Pullin, 2002; Villero et al., 2017). In this context, the availability of reliable information of the distribution of non-native species and the factors defining their patterns of distribution are essential for testing hypotheses and to generate robust predictive models (Ferrier et al., 2002; Jetz, Wilcove & Dobson, 2007).

With the development of Geographic Information System (GIS) tools, more precise data has become available for the management and analysis of spatial distributions of species (Jetz, Wilcove & Dobson, 2007). Specifically, the ecological niche modelling (ENM) is a geographically explicit approach that takes advantage of GIS tools to predict the past, present, and future distribution of species (Kozak, Graham & Wiens, 2008). Along with these predictions, this type of modeling is used to evaluate the role that different ecological factors have on distribution patterns (Elith et al., 2006; Elith, Kearney & Phillips, 2010; Lippitt et al., 2008; Davis, Malas & Minor, 2013; Madariaga et al., 2014). The ENM allows for the identification of sites that are highly suitable for populations and species based on their environmental requirements (Soberón & Nakamura, 2009; Elith, Kearney & Phillips, 2010). Unfortunately, most ENMs applied to marine habitats only use bioclimatic or oceanographic factors as predictive variables, which complicates the ability to test more complex hypotheses. However, recently, some studies in terrestrial systems have implemented post hoc analyses on ENM to include anthropic variables (Alaniz, Grez & Zaviezo, 2018). Thus, the inclusion of human activities as factors that affect the current distributions of the species could help to obtain more robust predictions about non-native species expansion (Zhu, Li & Zhao, 2017).

In the coasts of the Humboldt-current large marine ecosystem inhabits the invasive cryptogenic sea anemone *Anemonia alicemartinae* Häussermann & Försterra (2001) covering from the north (Arica, 18°S) to south-central of Chile (Concepción, 36°S) (Carlton, 1996). According to field reports, its distribution range has extended by approximately 1,928 km in the last 50 years (Häussermann & Försterra, 2001; Castilla et al., 2005). This species inhabits shallow intertidal and subtidal zones, as well as intertidal pools, and is capable of floating and disperse via currents (Häussermann & Försterra, 2001). Also, *A. alicemartinae* can adhere to a diverse array of substrates given its high capacity for adhesion and re-adhesion, a characteristic that also influences its dispersal (Häussermann & Försterra, 2001). López, Arancibia & Neill (2013) have suggested that *A. alicemartinae* has at least two dispersal mechanisms: short distance dispersal (within habitat), where the organism selects local conditions for settlement; and long-distance dispersal (among habitats), that could facilitate the colonization of new sites over large spatial scales. Therefore, one of the hypotheses suggests that its rapid expansion in the last several years may be explained by these particular biological traits (Häussermann & Försterra, 2001).

However, in addition to the high dispersal potential of *A. alicemartinae*, it has been proposed that the distribution expansion may be explained by human-mediated southward transport associated with regional-scale maritime activities (see Canales-Aguirre et al., 2015). Using molecular tools, Canales-Aguirre et al. (2015) show a lack of population genetic structure in *A. alicemartinae* along the southeastern pacific coast with greater genetic diversity at sites near main Chilean ports. These results strongly suggest that dispersal over large distances is likely also aided by anthropic vectors such as maritime activity at regional scales. The objective of this study was to evaluate the relative importance of environmental variables on the range expansion of *A. alicemartinae* along the southern coast of Chile using an ENM and its potential relationship with dispersion vectors such as maritime transport.

## Materials and Methods

### Study area and presence records of *A. alicemartinae*

**Figure 1:**
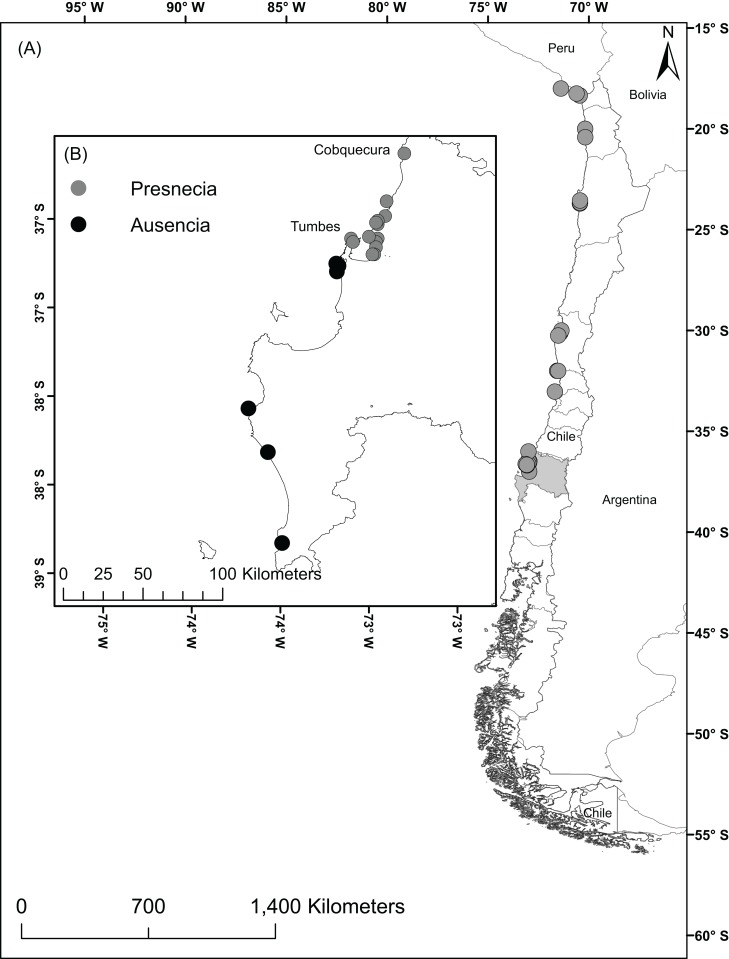
Localities with presence of the sea anemone *A. alicemartinae* along the Humboldt-current large marine ecosystem. (A) Locations where *A. alicemartinae* has been reported in previous studies, (B) Sampling study area in the Biobío region; the sites with presence of *A. alicemartinae* are shown by gray circles and black circles represent sites where *A. alicemartinae* was not present.

The study area comprised the Humboldt-current large marine ecosystem coasts from Perú to the southern zones of Chile including the Humboldtian, Central Chile, and Araucanian ecoregions (Spalding et al., 2007). These regions encompass the currently known geographic distribution of *A. alicemartinae*. In the year 2014, we surveyed the intertidal zone of 21 sites through directed searching for 2 h. To complement the field data, historical records were also used for 13 additional sites (Häussermann & Försterra, 2001; Canales-Aguirre et al., 2015). These 34 localities were haphazardly chosen in the way to represent the three ecoregions where *A. alicemartinae* has been reported, and to include port and no-port areas. The total dataset includes 27 georeferenced sites with presence of *A. alicemartinae* (Fig. 1A). GBIF records were also considered for this analysis, but after filtering for redundancy, spatial independence, and misclassified terrestrial locations, there were only 17 records which did not add to our analyses.

### Environmental database

The oceanographic variables used for analyses were obtained from Marspec database (Sbrocco & Barber, 2013). We downloaded 11 variables: Concavity (degrees), Mean Annual SSS (psu), SSS of the month with the lowest salinity (psu), SSS of the saltiest month (psu), Annual range in SSS (psu), Annual variance in SSS (psu), Mean Annual SST (°C), SST of the coldest month (°C), SST of the warmest month (°C), Annual range in SST (°C), Annual variance in SST (°C) at a spatial resolution of 30 arcseconds (∼1 Km). This database has a higher spatial resolution of data compared to other marine databases, such as BioOracle, which presents a resolution of 5 arcminutes (http://www.oracle.ugent.be/, Tyberghein et al., 2012). The data processing was run in ArcGis 10.3 (Esri, Redlands, CA, USA).

### Ecological niche modeling

To evaluate the potential distribution of *A. alicemantinae* based on oceanographic predictors we used the algorithm Maxent (Phillips, Anderson & Schapire, 2006) through the “dismo package” version 1.1-4 (Hijmans et al., 2017). We performed process selected oceanographic variables. Using the variance inflation factor (VIF) to evaluate the collinearity between predictors. A VIF > 10 is a sign of collinearity problems (Quinn & Keough, 2002). Four uncorrelated variables were finally selected to run the ENM: sea surface salinity of the saltiest month, annual variance in sea surface salinity, mean annual sea surface temperature, and annual variance in sea surface temperature.

To take into account possible sampling biases in the records of *A. alicemartinae* caused by oversampling in some areas, we estimated a Kernel density of the current records to generate a density surface. This density surface represents the magnitude per unit area of the points with high values at the location of the point and decreasing as distance increases (Silverman, 1986). The background points were generated on this density surface using the randomPoint function in the “dismo” package (Hijmans et al., 2017), and the “Probability” argument, in which the values of the mask represent a weight of the probability.

Given the low number of records (27 localities recorded) we used the Jackknife resampling technic for partition of the occurrence data (Pearson et al., 2007; Peterson et al., 2011; Shcheglovitova & Anderson, 2013), as a mean to evaluate the predictive ability of the model. Given that this technique requires independence in the occurrence data, we first evaluated the spatial autocorrelation of the records through the Moran I index in the SAM software (Rangel, Diniz-Filho & Bini, 2010). No spatial autocorrelation was detected (Moran I: 0.107; SD = 0.2; *p* = 0.693, Table S1).

### Model selection and ENM evaluation

Ecology niche model alternative models were generated by considering four uncorrelated predictors, configuring the parameters of the feature classes (FCs) and the regularization multipliers (RM; between 0.5 and 1 with a step value of 0.5). FCs determine the potential shape of the response curves (e.g., linear, quadratic; see Elith et al., 2011; Shcheglovitova & Anderson, 2013). The constraints imposed by features result in models of varying complexities; for example, models built with linear features are less complex than quadratic models (Shcheglovitova & Anderson, 2013). On the other hand, RM values impose penalties on the complexity of the model, where high RM values decreases the chance that the model could become overly complex or overfit (i.e., a protection against overfitting) (Shcheglovitova & Anderson, 2013). Multiple models were generated by varying the features class and RM.

The models were selected through the akaike information criterion (AIC; the lowest AIC value) which is a measure of relative adjustment, proportional to the likelihood of the model and the number of parameters used (Akaike, 1974; Burnham & Anderson, 2002). The best fit model was represented on the geographical space, where zero indicates minimum suitability and one indicates maximum suitability. The selection of models was made through the package ENMeval (Muscarella et al., 2014). Model constructions and analyses were run in the R software (R Core Team, 2017).

To evaluate the role of the human activity in the geographic range expansion of *A. alicemartinae*, the number of ship landings in ports was considered. These data were obtained from the Maritime Statistics Bulletin of the Dirección General del Territorio Marítimo y Marina Mercante from Chile (DIRECTEMAR; http://web.directemar.cl/estadisticas/maritimo/default.htm) and from the National Port Authority of Perú (https://www.apn.gob.pe). Data were digitalized and the coverage was determined through interpolation with weighted reverse distances, both using the software *ArcGIS 10.2* (ESRI, Redlands, CA, USA).

We performed a spatial regression analysis between the geographical distribution of *A. alicemartinae* predicted by the ENM and the layer of human activities, using simultaneous autoregressive models (SAR); SAR has shown a greater performance when dealing with data that presents autocorrelation (Dormann et al., 2007; Tognelli & Kelt, 2004). The autoregressive analyzes were performed in the SAM 4.0 software (Rangel, Diniz-Filho & Bini, 2010).

We uploaded all the data sets to Zenodo (https://zenodo.org/record/2641259#.XLTwQphKiUk). We included all the variables used in *.asc format file. Also, the R scripts were let available.

## Results

The field sampling of *A. alicemartinae* in the 21 sampled sites allowed the incorporation of 15 new records that had not been previously registered of this species between the intertidal rocky shore of Cobquecura (36°S, 72.96°W) and Tumbes (36.60°S, 73.40°W). This result extended the known southern distribution of this species by approximately 50 km from the intertidal rocky shore of Concepción (36°S) to Tumbes (36.60°S) (Fig. 1B).

**Figure 2:**
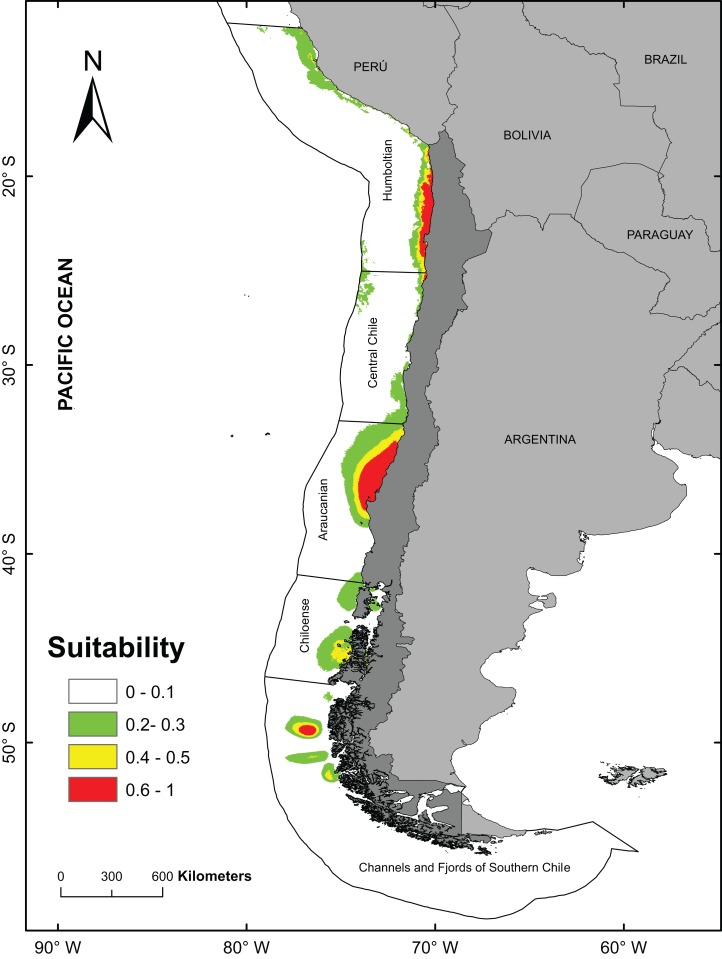
Potential distribution of *Anemonia alicemartinae* predicted by the best Ecological Niche Model. Analyses were based on oceanographic (chlorophyll, nitrate, salinity, and sea surface temperature) and anthropogenic variables (number of landings of ships in ports).Values are in log scale, where 0 indicates minimum suitability and 1 indicates maximum suitability.

The best model (from a total of 40 models) to predict the fundamental niches had an area under curve = 0.888 and a AICc values of 736.6 (see Table 1; Table S2). For subsequent analyzes, the model with FC = Linear + Quadratic and RM = 1.5 (see Table S2) was used to obtain the final prediction on the potential distribution of *A. alicemartinae* (Table 1). The variables that mainly contributed to the predicted distribution of *A. alicemartinae* were annual variance in sea surface salinity (39.65%) and annual variance in sea surface temperature (30.5%). The model indicated high suitability of presence (range 60–100%) in many sites along the Humboldt-current large marine ecosystem coasts, including southernmost sites at 53°S where this species is not currently recorded (e.g., Palena, Chiloé, Magallanes; Fig. 2). The areas with the highest suitability included the coasts of the Biobío region (Bahía de Concepción) and the inland sea of Chiloé.

**Table 1:** Models with lowest AIC_corrected_ for the study area of Chile-Perú.

Models number	Response type (features)	Regularization multiplier	Full.AUC	AICc	delta.AICc	w.AIC	Parameters
**6**	**LQ**	**1.5**	**0.888**	**736.64**	**0.0**	**0.6**	**5**
4	LQ	1	0.886	739.85	3.2	0.1	7
1	L	0.5	0.893	740.40	3.8	0.1	5
8	LQ	2	0.891	741.62	5.0	0.1	5
7	L	2	0.892	742.01	5.4	0.0	3
5	L	1.5	0.893	742.51	5.9	0.0	4
3	L	1	0.893	743.18	6.5	0.0	5
9	L	2.5	0.891	744.57	7.9	0.0	3
2	LQ	0.5	0.885	745.62	9.0	0.0	9

**Notes:**

The best model is indicated in bold. Delta AIC, absolute difference between the lowest AICc and each AICc. wAIC is the weighted AIC which represents the “weight of evidence” in favor of model i being the best approximating model in the set (Burnham & Anderson, 2002). The model with the highest AIC weight represents the best model in the set.

L, Linear; LQ, Linear+Quadratic.

The regression analysis between the prediction of the best suitability model and the number of landings, as a proxy for anthropogenic activity, indicated a positive and significant relationship between both variables (OLS, *m* = 0.143, *p* < 0.01; SAR, *m* = 0.133, *p* < 0.01; Table 2).

**Table 2:** Relationship between the best suitability niche ecological model of *Anemonia alicemartinae* and the number of landings.

	OLS regression					SAR regression				
	Coeff	*r*	*R*^2^	*p*-value	AIC	Coeff	*r*	*p*-*R*^2^	*p*-value	AIC
Constant	−0.932	0.243	0.059	<0.01	−673.293	−0.86	0.23	0.053	<0.01	−664.569
Number of landings	0.143					0.133				

**Note:**

Relationship between the best suitability niche ecological model of *Anemonia alicemartinae* and the number of landings. Summary of the ordinary least squares (OLS) and simultaneous autoregressive model (SAR) regressions.

## Discussion

In the present study, using an ENM approach, we demonstrate that both annual variation in salinity and sea surface temperature are the main oceanographic factors that explain the distribution of the invasive cryptogenic sea anemone *A. alicemartinae* along the Humboldt Current coastal ecosystem. Temperature and salinity are important variables that affect physiological performance, reproduction, and behavior of marine invertebrate organisms at different life cycle stages (Spivak & Cuesta, 2009; Tittensor et al., 2010; Smyth, Mazik & Elliott, 2014). In particular, salinity has been described as one of the key limiting factors for the propagation of marine organisms, especially in sessile osmoconformers, since they are unable to actively adjust their extracellular osmolarity (Bonsdorff, 2006; Guppy & Withers, 1999). Podbielski et al. (2016) suggests that salinity may help to predict the expansion potential of the sea anemone *Diadumene lineata* in the Baltic Sea. They found that although this species may tolerate salinity >10 ppm, lower salinities restrict distribution expansion.

The positive and significant relationship between the geographical distribution predicted by the ENM and the number of landings of ships in ports, suggests that this anthropogenic factor contributes significantly to explain the current southernmost distribution of *A. alicemartinae*. This could be extended to sites in Patagonia that include the Chiloense ecoregion and the ecoregion of the channels and fjords of southern Chile (Spalding et al., 2007). These areas have high maritime traffic (ports, ships, DIRECTEMAR 2015, http://web.directemar.cl/estadisticas/maritimo/default.htm) that could facilitate the future dispersal and successful establishment of this species.

Previous studies of *A. alicemartinae* (Häussermann & Försterra, 2001; Canales-Aguirre et al., 2015) have not conclusively determined whether this species is native to northern Chile and has expanded its range southward, or if it is an introduced species invading the Chilean coast. However, given its conspicuous features, such as its coloration (intense red) and habitat use in the intertidal and subtidal zones, it is unlikely that this species has gone unnoticed in the records of native marine biota since the beginning of the 20th century (Häussermann & Försterra, 2001). These antecedents are in concordance with the sampling that we carried out for this study in the Biobío region, where a high abundance of *A. alicemartinae* was found (more than 30 ind/m^2^ in some localities), being easily identified by its coloration and recognizable morphological features, such as bipartite margin accretions and frequent longitudinal fission marks (Häussermann & Försterra, 2001). Therefore these observations support that *A. alicemartinae* is a recent invader from the Northern part of the Humboldt-current large marine ecosystem coast.

Our results show that maritime transport is an important factor to explain the distribution range of *A. alicemartinae*, which strengthens the hypothesis by Canales-Aguirre et al. (2015) that explains the higher genetic diversity observed in localities near ports. The use of ships as transportation vectors by this species can allow colonization by individuals from different population sources, increasing local genetic diversity. This pattern has been observed in other invasive invertebrate marine species, such as sponges (e.g., *Paralecilla magna*; Guardiola, Frotscher & Uriz, 2016), shrimps (e.g., *Palaemon macrodactylus*; Lejeusne et al., 2014) and barnacles (e.g., *Chthamalus proteus*; Zardus & Hadfield, 2005). These three examples show an increase in genetic diversity in recently invaded localities, which may be caused by repeated introduction events from different source populations that can compensate for the effects of genetic bottlenecks and restore equivalent or even higher levels of diversity than those observed in native populations (Zardus & Hadfield, 2005; Lejeusne et al., 2014; Guardiola, Frotscher & Uriz, 2016). Additional evidence that supports the high dispersal capacity of *A. alicemartinae* and the potential use of maritime transport as a spreading mechanism is the null impact of biogeographical barriers in this species. Along the Chilean coast, there are three biogeographic units: Peruvian Province, Intermediate Area, and Magellanic Province, determining two main biogeographic breaks at 30°S and 41–43°S (Camus, 2001; Hernández et al., 2005; Hormazabal, Shaffer & Leth, 2004). The present distribution of *A. alicemartinae* crosses the northern biogeographic break (30°S) that separates the Peruvian Province and the Intermediate Area. This break has been explained by the existence of coastal oceanographic features, such as strong kinetic eddies and patterns of sea surface temperatures, as well as an abrupt continental narrowing (Strub et al., 1998; Camus, 2001; Hormazabal, Shaffer & Leth, 2004). In several marine species, a biogeographic and phylogeograhic concordance is observed at this break, suggesting a limitation for genetic flow between both sides of the break (Haye et al., 2014; Sánchez et al., 2011). The lack of population genetic structure found by Canales-Aguirre et al. (2015) in *A. alicemartinae* along its whole distributional range evidences that this species may cross the 30°S biogeographic break maintaining a high connection between the northern and southern populations. The ability of this sea anemone to detach and reattach from different substrates, and the potential use of ships as dispersal vectors, would allow *A. alicemartinae* to colonize new places. Actually, it has been reported that maritime transport facilitates the crossing of natural boundaries in the ocean by species (Kaluza et al., 2010; Molnar et al., 2008). For example, a study carried out in the ballast water and hulls of 186 vessels in the North Sea found that 57% of the species recorded were non-native (Gollasch, 2002). Similar studies conducted in the coast of Chile revealed the presence of the invasive ascidian *Asterocarpa humilis* attached to hulls of international ships, which may explain the rapid spread of this species for more than 2,000 km along the coast (Pinochet et al., 2017).

Our estimations predict an expansion of the actual geographic range of *A. alicemartinae* toward southernmost regions of the south eastern pacific coast, explained by ecological attributes of the species and the dispersion vector (ships). Although there is little evidence that this species may impact native species survival or distribution, the increase of its geographic range toward austral localities could generate negative interactions with native species through interspecific competition, or prevent the recruitment of other species (preemptive competition).

There are few examples of works that use niche models to study possible expansions of the range of marine invertebrate invaders on the southeast coast. One study was carried out with a mechanical niche model approach to evaluate possible range expansions in the invaders *Ciona intestinalis* (now *Ciona robusta* in Chile, Bouchemousse, Bishop & Viard, 2016) and *Codium fragile* (Madariaga et al., 2014). The main results showed that both species can be propagated to most regions of the Chilean coast, which may affect diversity and community structure (Drouin, McKindsey & Johnson, 2011; Cordell, Levy & Toft, 2013). In another study, Januario et al. (2015) modeled and validated the environmental conditions that allow the persistence and propagation of the species complex *Ciona sp* A and B (now *Ciona intestinalis* and *Ciona robusta*, respectively) in the Chilean coast. They found that suitable areas for *Ciona intestinalis* are located between 30° and 40°S, meanwhile, areas around 45°S are the most appropriated for *Ciona robusta*. All these studies considered only environmental suitability to predict expansion range, however, other factors may interact to promote the propagation of these species. For example, in *Ciona intestinalis*, the potential capacity to attach to the hulls of ships may facilitate long-distance transport in this species (Sargent et al., 2013).

Finally, our results highlight the influence of anthropogenic variables affecting the distribution of marine invasive species, specifically maritime transport, indicating important implications for the conservation of biodiversity. For future studies, we recommend conducting laboratory and field experiments to evaluate the physiological tolerance and competition ability of *A. alicemartinae*. An integrative approach to modeling invasion dynamic of this species should include anthropogenic variables in conjunction with biotic and abiotic variables, which may affect or prevent the process of invasion to the Humboldt-current large marine ecosystem coast.

## Conclusions

Studying the factors determining geographical distribution of species, and then modeling and predicting under different scenarios, are steps of fundamental importance to generate policies for the management and conservation of biodiversity, especially when the introduction of non-native species has been increasing in the last decades. In this work, we showed the importance of the variability of salinity and temperature, coupled with anthropogenic factors such as maritime transport, as the main predictors of the range expansion of *A. alicemartinae*, a species with high dispersive and invasive potential, along the Chilean coast. Also, we showed that the most susceptible areas to invasion were those with high maritime activity and high variability of temperature and salinity. The expansion predicted by the ENM includes two southern ecoregions, reaching the Patagonian area, and covering most of the Humboldt-current large marine ecosystem.

## Supplemental Information

10.7717/peerj.7156/supp-1 Supplemental Information 1 Spatial autocorrelation analysis on data records of *Anemonia alicemartinae* through the Moran I index in the SAM software. Values of global Moran’s I statistics for each class of distance. Count=number of connections, DistCntr= maximum distance, Moran’s I= Moran’s index, P= p value, I (max)= maximum possible value of Moran’s, and I/I(max)= relative value of MoranM-BM-4s.

10.7717/peerj.7156/supp-2 Supplemental Information 2 Summary of candidate models assessed and tested using an Ecological Niche Modelling approach in *Anemonia alicemartinae*. Models are ordered from lowest to highest AIC values.

10.7717/peerj.7156/supp-3 Supplemental Information 3 Localities with presence of A. alicemartinae. For each locality with presence of *A. alicemartinae* the geographic coordinates (latitude and longitude) are presented.

10.7717/peerj.7156/supp-4 Supplemental Information 4 R script for niche model analyses. The script contains the commands to calibrate and evaluate different ecological niche models in the package ENMeval.

10.7717/peerj.7156/supp-5 Supplemental Information 5 Environmental and anthropic variables. The link contains the oceanographic variables used to build the Ecological Niche Models with a resolution of 1 km. Also, a data layer with the number of ships landings per port is included.

## Additional Information and Declarations

### Data Availability

The following information was supplied regarding data availability: Additional data is available at Zenodo: Reinaldo Rivera Jara. (2019). Spread of the non-native anemone *Anemonia alicemartinae* Häussermann & Försterra (2001) along the Humboldt-current large marine ecosystem: an ecological niche model approach (Version 1) [Data set]. Zenodo. http://doi.org/10.5281/zenodo.2641259. This dataset contains the oceanographic variables used to build the Ecological Niche Models with a resolution of 1 km. Also, a data layer with the number of ships landings per port is included.
