# Supplementary material for: Spread of the non-native anemone Anemonia alicemartinae Häussermann & Försterra, 2001 along the Humboldt-current large marine ecosystem: an ecological niche model approach
Source: PeerJ. 2019 Jul 4;7:e7156. doi: 10.7717/peerj.7156 (PMC6612420; doi:10.7717/peerj.7156)
Supplement: Supplemental Information 1 — Values of global Moran’s I statistics for each class of distance. Count=number of connections, DistCntr= maximum distance, Moran’s I= Moran’s index, P= p value, I (max)= maximum possible value of Moran’s, and I/I(max)= relative value of MoranM-BM-4s. [file peerj-07-7156-s001.docx]

Table. S1. Values of global Moran's I statistics for each class of distance. Count= number of connections, DistCntr= maximum distance, Moran’s I= Moran’s index, P= p value, I (max)=maximum possible value of Moran’s, and I/I(max)=relative value of Moran´s.

| D.Class | Count | DistCntr | Moran's I | P | I (max) | I/I(max) |
| --- | --- | --- | --- | --- | --- | --- |
| 1 | 34 | 3.175 | 0.005 | 0.995 | 1.664 | 0.003 |
| 2 | 34 | 8.15 | 0.09 | 0.688 | 2.176 | 0.041 |
| 3 | 32 | 14.647 | -0.107 | 0.668 | 1.345 | -0.08 |
| 4 | 34 | 23.205 | -0.175 | 0.372 | 2.304 | -0.076 |
| 5 | 32 | 52.837 | 0.152 | 0.437 | 1.904 | 0.08 |
| 6 | 34 | 204.942 | -0.23 | 0.261 | 1.133 | -0.203 |
| 7 | 32 | 381.082 | 0.048 | 0.789 | 1.392 | 0.035 |
| 8 | 34 | 478.527 | 0.115 | 0.432 | 1.851 | 0.062 |
| 9 | 32 | 568.676 | 0.411 | 0.065 | 1.977 | 0.208 |
| 10 | 34 | 672.174 | -0.114 | 0.588 | 1.067 | -0.107 |
| 11 | 34 | 731.104 | -0.098 | 0.623 | 1.239 | -0.079 |
| 12 | 32 | 833.701 | -0.076 | 0.673 | 2.079 | -0.037 |
| 13 | 34 | 1118.914 | 0.221 | 0.332 | 0.992 | 0.223 |
| 14 | 32 | 1379.64 | -0.278 | 0.261 | 1.15 | -0.242 |
| 15 | 34 | 1453.469 | -0.433 | 0.05 | 1.926 | -0.225 |
| 16 | 32 | 1494.687 | -0.194 | 0.302 | 2.188 | -0.089 |
| 17 | 34 | 1675.404 | 0.084 | 0.643 | 2.102 | 0.04 |
| 18 | 32 | 1912.622 | -0.241 | 0.261 | 1.07 | -0.225 |
| 19 | 34 | 2035.639 | -0.277 | 0.191 | 0.987 | -0.281 |
| 20 | 34 | 2065.451 | 0.252 | 0.181 | 2.061 | 0.122 |
