# Supplementary material for: Spread of the non-native anemone Anemonia alicemartinae Häussermann & Försterra, 2001 along the Humboldt-current large marine ecosystem: an ecological niche model approach
Source: PeerJ. 2019 Jul 4;7:e7156. doi: 10.7717/peerj.7156 (PMC6612420; doi:10.7717/peerj.7156)
Supplement: Supplemental Information 2 — Models are ordered from lowest to highest AIC values. [file peerj-07-7156-s002.docx]

Table. S2. Summary of candidate models assessed and tested. Ordered models of lower AIC value at higher value.

| Response type (features) | Regularization multiplier | train.AUC | avg.test.AUC | var.test.AUC | avg.diff.AUC | var.diff.AUC | AICc | delta.AICc | w.AIC | parameters |
| --- | --- | --- | --- | --- | --- | --- | --- | --- | --- | --- |
| L | 0.5 | 0.893 | 0.888 | 0.466 | 0.061 | 0.279 | 740.399 | 3.762 | 0.093 | 5 |
| LQ | 0.5 | 0.885 | 0.871 | 0.824 | 0.081 | 0.540 | 745.623 | 8.987 | 0.007 | 9 |
| L | 1 | 0.893 | 0.888 | 0.467 | 0.061 | 0.278 | 743.175 | 6.538 | 0.023 | 5 |
| LQ | 1 | 0.886 | 0.875 | 0.716 | 0.075 | 0.448 | 739.847 | 3.210 | 0.122 | 7 |
| L | 1.5 | 0.893 | 0.888 | 0.478 | 0.061 | 0.285 | 742.505 | 5.869 | 0.032 | 4 |
| **LQ** | **1.5** | **0.888** | **0.878** | **0.634** | **0.070** | **0.401** | **736.636** | **0.000** | **0.610** | **5** |
| L | 2 | 0.892 | 0.887 | 0.487 | 0.062 | 0.289 | 742.006 | 5.370 | 0.042 | 3 |
| LQ | 2 | 0.891 | 0.882 | 0.594 | 0.067 | 0.384 | 741.617 | 4.981 | 0.051 | 5 |
| L | 2.5 | 0.891 | 0.886 | 0.498 | 0.063 | 0.295 | 744.571 | 7.935 | 0.012 | 3 |
| LQ | 2.5 | 0.897 | 0.886 | 0.551 | 0.063 | 0.368 | 747.491 | 10.855 | 0.003 | 5 |
| L | 3 | 0.890 | 0.884 | 0.519 | 0.064 | 0.306 | 747.448 | 10.812 | 0.003 | 3 |
| LQ | 3 | 0.898 | 0.889 | 0.530 | 0.061 | 0.359 | 747.877 | 11.241 | 0.002 | 4 |
| L | 3.5 | 0.889 | 0.881 | 0.558 | 0.067 | 0.328 | 750.662 | 14.026 | 0.001 | 3 |
| LQ | 3.5 | 0.898 | 0.890 | 0.533 | 0.061 | 0.362 | 750.650 | 14.013 | 0.001 | 4 |
| L | 4 | 0.887 | 0.878 | 0.605 | 0.070 | 0.353 | 754.242 | 17.606 | 0.000 | 3 |
| LQ | 4 | 0.897 | 0.889 | 0.536 | 0.061 | 0.361 | 753.722 | 17.086 | 0.000 | 4 |
| L | 4.5 | 0.885 | 0.873 | 0.663 | 0.073 | 0.382 | 758.215 | 21.579 | 0.000 | 3 |
| LQ | 4.5 | 0.896 | 0.887 | 0.550 | 0.062 | 0.368 | 754.153 | 17.516 | 0.000 | 3 |
| L | 5 | 0.880 | 0.864 | 0.743 | 0.078 | 0.420 | 762.610 | 25.974 | 0.000 | 3 |
| LQ | 5 | 0.893 | 0.885 | 0.567 | 0.063 | 0.372 | 757.677 | 21.041 | 0.000 | 3 |
| L | 5.5 | 0.874 | 0.851 | 0.848 | 0.083 | 0.478 | 767.447 | 30.811 | 0.000 | 3 |
| LQ | 5.5 | 0.891 | 0.882 | 0.596 | 0.066 | 0.385 | 761.559 | 24.923 | 0.000 | 3 |
| L | 6 | 0.864 | 0.830 | 1.000 | 0.092 | 0.544 | 772.736 | 36.100 | 0 | 3 |
| LQ | 6 | 0.888 | 0.877 | 0.646 | 0.069 | 0.404 | 765.829 | 29.193 | 0.000 | 3 |
| L | 6.5 | 0.850 | 0.794 | 1.289 | 0.107 | 0.662 | 778.472 | 41.836 | 0 | 3 |
| LQ | 6.5 | 0.884 | 0.867 | 0.736 | 0.074 | 0.449 | 770.515 | 33.879 | 0 | 3 |
| L | 7 | 0.832 | 0.746 | 1.746 | 0.129 | 0.787 | 784.627 | 47.991 | 0 | 3 |
| LQ | 7 | 0.875 | 0.847 | 0.887 | 0.084 | 0.525 | 775.643 | 39.007 | 0 | 3 |
| L | 7.5 | 0.806 | 0.738 | 1.850 | 0.128 | 0.797 | 791.153 | 54.517 | 0 | 3 |
| LQ | 7.5 | 0.861 | 0.812 | 1.193 | 0.099 | 0.670 | 781.223 | 44.587 | 0 | 3 |
| L | 8 | 0.758 | 0.738 | 1.859 | 0.129 | 0.803 | 797.975 | 61.339 | 0 | 3 |
| LQ | 8 | 0.834 | 0.755 | 1.669 | 0.128 | 0.798 | 787.251 | 50.615 | 0 | 3 |
| L | 8.5 | 0.746 | 0.737 | 1.872 | 0.130 | 0.811 | 797.794 | 61.157 | 0 | 2 |
| LQ | 8.5 | 0.795 | 0.742 | 1.826 | 0.127 | 0.788 | 793.692 | 57.056 | 0 | 3 |
| L | 9 | 0.747 | 0.735 | 1.896 | 0.132 | 0.820 | 798.528 | 61.892 | 0 | 2 |
| LQ | 9 | 0.749 | 0.741 | 1.839 | 0.128 | 0.792 | 796.682 | 60.045 | 0 | 2 |
| L | 9.5 | 0.747 | 0.733 | 1.935 | 0.135 | 0.834 | 799.374 | 62.738 | 0 | 2 |
| LQ | 9.5 | 0.748 | 0.739 | 1.857 | 0.129 | 0.797 | 797.359 | 60.723 | 0 | 2 |
| L | 10 | 0.746 | 0.731 | 1.988 | 0.137 | 0.849 | 800.347 | 63.711 | 0 | 2 |
| LQ | 10 | 0.746 | 0.738 | 1.898 | 0.131 | 0.809 | 798.143 | 61.507 | 0 | 2 |

train.AUC =training AUC, avg.test.AUC=mean of the k test AUCs, var.test.AUC= variance of the k test AUCs, avg.diff.AUC =mean of all differences between the k training and test AUCs, var.diff.AUC= variance of all differences between the k training and test AUCs, AIC= Akaike information criteria, delta.AICc=absolute difference between the lowest AICc and each AICc, w.AIC: AIC weight, calculated as the average relative model likelihood (exp(-0.5 * delta.AICc)) across all models (Burnham and Anderson 2002). the best model is marked with black color.

L=Linear; LQ=Linear+Quadratic
