# Supplementary material for: Spread of the non-native anemone Anemonia alicemartinae Häussermann & Försterra, 2001 along the Humboldt-current large marine ecosystem: an ecological niche model approach
Source: PeerJ. 2019 Jul 4;7:e7156. doi: 10.7717/peerj.7156 (PMC6612420; doi:10.7717/peerj.7156)
Supplement: Supplemental Information 5 — The link contains the oceanographic variables used to build the Ecological Niche Models with a resolution of 1 km. Also, a data layer with the number of ships landings per port is included. [file peerj-07-7156-s005.docx]

Metadata: Environmental and anthropic variables.

The following link contains the oceanographic variables used to build the Ecological Niche Models with a resolution of 1 km. Also, a data layer with the number of ships landings per port is included.

**https://zenodo.org/record/2562092#.XGRzoTNKiUk**
